# Phenotypic Characterization of Postharvest Fruit Qualities in Astringent and Non-astringent Persimmon (*Diospyros kaki*) Cultivars

**DOI:** 10.3389/fgene.2021.670929

**Published:** 2021-06-07

**Authors:** Akhilesh Yadav, Anton Fennec, Changfei Guan, Yong Yang, Bettina Kochanek, David Israel, Anat Izhaki, Shmuel Zilkah, Haya Friedman

**Affiliations:** ^1^Department of Postharvest Science of Fresh Produce, Agricultural Research Organization (ARO)-Volcani Institute, Rishon LeZion, Israel; ^2^State Key Laboratory of Crop Stress Biology for Arid Areas, College of Horticulture, Northwest A&F University, Yangling, China; ^3^Department of Fruit Tree Sciences, Agricultural Research Organization (ARO)-Volcani Institute, Rishon LeZion, Israel

**Keywords:** astringency, *Alternaria alternata*, firmness, I*_*AD*_*, cracks

## Abstract

Phenotypic characterization of postharvest traits is essential for the breeding of high-quality fruits. To compare postharvest traits of different genetic lines, it is essential to use a reference point during fruit development that will be common to all the lines. In this study, we employed a non-destructive parameter of chlorophyll levels to establish a similar physiological age and compared several postharvest traits of ten astringent and seven non-astringent persimmon cultivars. The fruit’s traits examined were astringency, weight, total soluble solids (TSS), titratable acidity (TA), chlorophyll levels (I*_*AD*_*), color (hue), firmness, color development and firmness loss during storage, crack development, and susceptibility to *Alternaria* infection. Although the chlorophyll (I*_*AD*_*) index and color (hue) showed a high correlation among mature fruits of all cultivars, the chlorophyll parameter could detect higher variability in each cultivar, suggesting that I*_*AD*_* is a more rigorous parameter for detecting the developmental stage. The average weight, TSS, and TA were similar between astringent and non-astringent cultivars. Cracks appeared only on a few cultivars at harvest. Resistance to *Alternaria* infection and firmness were lower in astringent than in non-astringent cultivars. Only the astringent cultivar “32” was resistant to infection possibly due to the existence of an efficient peel barrier. It was concluded that a high correlation existed between astringency, susceptibility to *Alternaria* infection, and firmness. Cracks did not correlate with astringency or firmness. The phenotypic traits evaluated in this work can be used in future breeding programs for elite persimmon fruits.

## Introduction

Persimmon fruits are berries of deciduous trees. Cultivated persimmons (*Diospyros kaki*) are classified into four groups, based on the flesh color affected by pollination, and on the astringency loss pattern. The types are: pollination constant non-astringent (PCNA), pollination variant non-astringent (PVNA), pollination constant astringent (PCA), and pollination variant astringent (PVA) (Sato and Yamada, 2016). Astringency is the oral puckering/dry sensation resulting from tannin consumption (Ekambaram et al., 2016). Most of the persimmon cultivars are astringent, belonging to the PVA or PCA types, and originated from Japan, Korea, and China, and they share genetic similarity to European cultivars (Yonemori et al., 2008). The genetic similarity between different Chinese local accession of *D. kaki* has been documented (Yonemori et al., 2008; Guan et al., 2019). Breeding programs of persimmon mainly in China and Japan emphasized the development of non-astringent cultivars, and less attention was given to postharvest traits (Guo and Luo, 2006; Sato and Yamada, 2016; Zhang et al., 2016).

Traditionally, in its original countries, the persimmons are consumed soft, when the fruit lost its astringency. In the Japanese non-astringent cultivars, proanthocyanidins stop to accumulate after 6–7 weeks of bloom, leading to a non-astringent phenotype. This is due to the expression arrest of genes involved in the flavonoid pathway (Ikegami et al., 2005). The genetic trait of the astringent/non-astringent (AST/ast) is controlled by a single recessive locus (Akagi et al., 2011). However, the exact genetic modification responsible for the non-astringent phenotype within the AST locus has not been identified. This genetic modification reduced the accumulation of polyphenols and condensed tannins (Yadav et al., 2021a).

In many western countries mainly in Europe, the fruit is consumed firm and protocols have been developed to remove astringency (Ben Arieh and Sonego, 1993; Arnal and Del Río, 2003). Long-term storage at low temperatures is a common strategy in fruit preservation (Lachman et al., 2000), however, in persimmon long-term storage of firm fruit causes firmness loss, and damage (Salvador et al., 2004; Yadav et al., 2021b). In addition, some cultivars are sensitive to the development of necrotic lesions often referred to as “Blackspot disease” (BSD) (Prusky et al., 1981), caused by *Alternaria alternata*. *Alternaria* infects the persimmon fruits by intrusion through small cracks prevalent under the sepal (Kobiler et al., 2011), and it remains dormant, forming a quiescent infection that develops during storage and with progression in ripening. In some seasons, as much as 30% of the leading persimmon cultivar “Triumph” in Israel is affected by *Alternaria* (Prusky et al., 1981).

Cracks on fruit surfaces can hinder the commercialization of fruit like apple (Ginzberg and Stern, 2019), pomegranate (Davarpanah et al., 2018), and cherry (Winkler and Knoche, 2019). They can also reduce persimmon quality and most of the Japanese persimmon cultivars including “Shinshu” (Yamada and Sato, 2003), and the cultivar “Triumph” suffers from cracking (Biton et al., 2014). The susceptibility of fruit to crack development is a major quality parameter of persimmon fruit (Onoue et al., 2016; Yadav et al., 2021b).

Previously, a study evaluated a large collection of persimmon cultivars for another set of traits which include total phenolic, vitamin C, carotenoids, glucose, fructose, and sucrose content (Giordani et al., 2011). However, to enable the marketing of the persimmon fruit, it is necessary to breed cultivars with high postharvest qualities, which exhibit high firmness, and with no blemishes or pathogen infection. Although the genus includes many species, only a few are commercially cultivated and their postharvest performance has rarely been examined.

QTL analysis for postharvest qualities has been implemented in several crops (Friedman, 2019), but rarely for persimmon. QTL analysis has to take into account the developmental stage of the fruit because it might affects the trait examined. Therefore, when comparing traits of different cultivars/accessions it is essential to determine that the physiological/developmental stage/age is similar in all the cultivars. Chlorophyll levels have been suggested as a suitable parameter to determine the physiological age in peaches and apples (Zude, 2003; Lurie et al., 2012; Lurie et al., 2013).

In Israel, persimmon cultivation started during the early 80s in Volcani Institute, and currently, there are 17 persimmon cultivars. Most of them are uncharacterized and very little is known about their fruit’s qualities. This work focused on the characterization of postharvest qualities of firm fruits of the different persimmon cultivars since firm fruits are desirable in commerce. It established the use of non-destructive chlorophyll determination to determine the harvest time. Using similar parameters of chlorophyll levels for all the cultivars provided a reference point that enabled the comparison of other traits like astringency, weight, TSS, TA, crack development, firmness, firmness loss, and color development during storage, as well as *Alternaria* susceptibility.

## Materials and Methods

### Plant Materials

All the persimmon fruits were harvested from 17 cultivars (Supplementary Table 1) between August to December from a 12-year-old orchard, located in Volcani Institute, Rishon Lezion, Israel (31° 59′ 25″ N, 34° 49′ 2.84″ E). The data presented in this study is for the years 2017 and 2019. Trees were planted 4 m apart in rows of 4 m apart and *D. virginiana* was used as a rootstock. Trees were drip irrigated at 0.5 m^3^/m^2^ containing N: P: K at 50:30:50 mg/L.

The fruits of each cultivar were collected during the season according to their ripening/maturity stage. Fruits at their Green-Yellow appearance of each cultivar were collected from spatially distributed five plots. Approximately 130–150 healthy and unblemished fruits were visually selected for further experiments.

### Determination of Total Soluble Solids (TSS) and Titratable Acidity (TA)

TSS and TA were determined in fruit juice. The juice was produced by pressing whole fresh fruit slices through sterile gauze. Measurements were done on 5 repetitions each of one fruit of each cultivar. TSS was determined with a digital Refractometer (PR-1, ATAGO Co., Ltd. Japan) and was expressed in Brix°. Titratable acidity was measured with an automated Titrator (set 2.730.0010. Ω Metrohm, Switzerland) by diluting 1 ml of fresh juice in 60 ml distilled water, and the results were expressed in Malic acid equivalent (g/L).

### Chlorophyll and Color Determination

Chlorophyll was measured with a DA-meter (TR-Turoni, Forli, Italy) that measures the index of absorbance difference (I*_*AD*_* = A670nm – A720nm) (Ziosi et al., 2008). Measurements were taken from 10 fruits of each cultivar; each fruit was measured at 4 places (2 from the peduncle side and 2 from the style side) and the average I*_*AD*_* was presented.

The color was determined with chroma meter CR-300 (Konica Minolta Inc, Tokyo, Japan), by measuring the color of 10 fruits at 4 spots on each fruit (Munera et al., 2017). All the color values were acquired in “L,” “C,” “H” color space (CIELAB model), and the results of “H” (hue) were presented.

### Determination of Astringency Index, and Firmness

**FIGURE 1:**
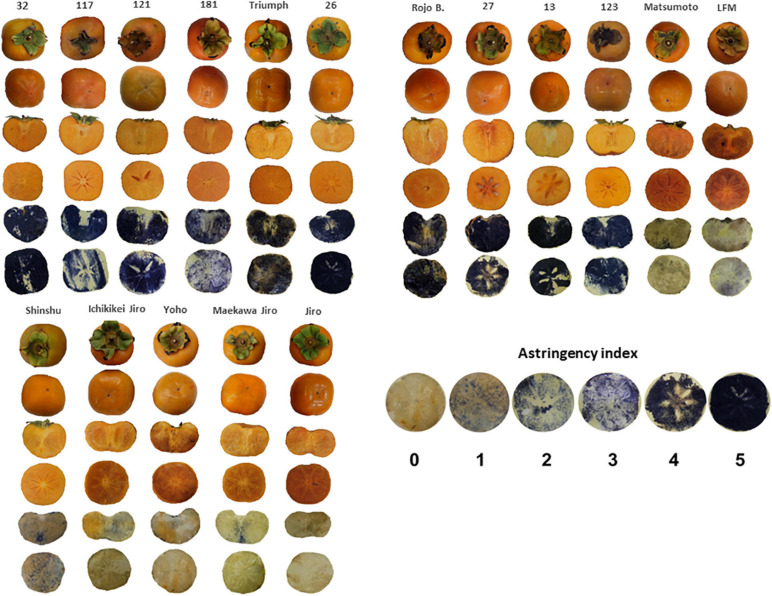
Representative pictures of the persimmon cultivars showing the shape, and astringency imprint. The astringency index scale represents the incrementing scale of astringency based on FeCl_3_ staining. Dark imprint represents high astringency (rank 5) and light color non-astringency (rank 0).

The astringency ranking was determined by the FeCl_3_ staining method, which was adopted by a commercial packing house (Mor Ha Sharon Fruits Ltd., Israel). FeCl_3_ paper was prepared by dipping filter paper in a solution of 5% FeCl_3_ (Sigma-Aldrich, Israel) until the paper became yellow and then dried and kept in darkness until further use. Fresh fruit slices obtained from 5 fruits of each cultivar were pressed against a FeCl_3_ paper for 30 s and then removed. The Astringency index was estimated based on a scale presented in Figure 1.

Fruit firmness was determined on 5–10 peeled fruits from each cultivar. An 8 mm diameter flat probe was used for the harvest 2017 using an automatic penetrometer (Agrosta14^®^ Motor V3, Agrosta, France) and a 5 mm diameter flat probe for the harvest 2019 using a texture analyzer (Stable Microsystem Ltd., United Kingdom). The results for both years were expressed in N/cm^2^.

### Cracks Evaluation

Cracks on the fruit surface were examined by staining with methylene blue (Zoffoli et al., 2008). The fruits were dipped in a solution of 0.1 mM methylene blue (Sigma-Aldrich, Israel). The crack index was evaluated by a scale from 0- no crack, 1 – 25% of the surface had cracks, 2 – 50%, 3 – 75%, and 4 – 100%. The crack index was calculated as described below. Cracks on the fruit surface were assessed on 5 fruits at harvest and after three months of storage at 0°C.

$$\mathrm{Crack}\,\mathrm{index}=\frac{\begin{array}{c} \Sigma \,\mathrm{fruit}\,\mathrm{index1}*1+\Sigma \,\mathrm{fruit}\,\mathrm{index2}*2+\\ \Sigma \,\mathrm{fruit}\,\mathrm{index3}*3+\Sigma \,\mathrm{fruit}\,\mathrm{index4}*4 \end{array}}{\mathrm{Total}\,\mathrm{fruit}\,\mathrm{number}}$$

### Preparation of *Alternaria* Conidia Suspension and Fruit Inoculation

*Alternaria alternata* was cultivated on PDA (Becton Dickinson, United States) containing 50 mg/ml Chloramphenicol (Formedium^TM^, England) in 90 mm × 15 mm petri dishes. The cultures were incubated at room temperature. Conidia from mature cultures were obtained by scraping the culture with a sterile spreader in a 1 ml solution of 0.03% Tween-20 in distilled water to prevent clumping. The suspended conidia were filtered and a final stock concentration was adjusted to 10^5^ spores/ml.

The fruits were disinfected with Taharsept (500 ppm, latent available chlorine-LAC) and set to dry. In the year 2017 inoculation was performed on pierced fruit, while in the year 2019 the inoculation was placed on an intact surface. Piercing was performed with a 2 mm needle, with a rubber stopper. Inoculation either on pierced or non-pierced fruit was performed by placing a 7 μl conidial suspension on 8 spaced-apart spots. Control fruits were treated with a sterile drop of 0.03% Tween-20 solution. The infection incidence (%; calculated by the number of infected spots out of inoculated spots) and the decay diameter (mm) was determined. In the pierced fruit (2017) the scoring was done following 7 days at room temperature and in the non-pierced fruit (2019), infected fruits were stored at 0°C for 3 months before scoring.

### Micro-Morphology of the Fruits Surface Under Scanning Electron Microscope (SEM)

SEM was used to examine the persimmon fruits’ surface. Sections were taken from the equatorial regions of the fruit and were fixed in Formalin-Acetic acid-Alcohol (FAA) solution containing 10% formalin (v/v from a stock solution of 35% formaldehyde), 5% glacial acetic acid (v/v), and 50% ethanol, (v/v from 95% ethanol) until analysis. Samples were dehydrated by serial dilution of ethanol. Following dehydration, the samples were dried in K850 critical point dryer (Quorum Technology Ltd., United Kingdom), followed by a coating with gold-palladium alloy using SC7620 mini sputter coater (Quorum Technology Ltd., United Kingdom). The samples were analyzed by benchtop SEM, JCM-600 (JEOL, Japan).

### Genetic Diversity by SSR Marker

The SSR primers were the same as reported (Guan et al., 2019; Guan et al., 2020) and modified with 3 fluorescent markers at the 5’-end, including FAM (6-carboxy-fluorescein), HEX (hexachloro-fluorescein), and TAMRA (carboxy tetramethyl-rhodamine). The UPGMA (unweighted pair group method with arithmetic mean) tree was performed on the DARwin software.^1^

### Statistics Analyses

All the statistical analyses were done using SAS JMP Pro 13.0.0 (2016). Analysis of Means (ANOM) (Mendeş and Yiğit, 2018) was conducted for I*_*AD*_* and color (hue) results of all the cultivars. This analysis took into account all the data points from each cultivar. The average line represents the values of all the data points. The upper and lower decision limits (UDL and LDL) are the values for each cultivar that above or below that, respectively, deviates from the average at *p* ≤ 0.001. All the one-way ANOVA tests and the different letters of significance were acquired by the Tukey-Kramer HSD test at *p* ≤ 0.05 following a normal distribution test. Correlations were established by means of the Pearson correlation coefficient (R). ClustVis online tool^2^ was used to create a heat-map for all parameters of 2017 and 2019. The data for the heat map was normalized as a percentage of the average values of each parameter.

## Results

### Harvest Dates, Characterization of Astringency, TSS, TA and Genetic Diversity of the Different Cultivars

**FIGURE 2:**
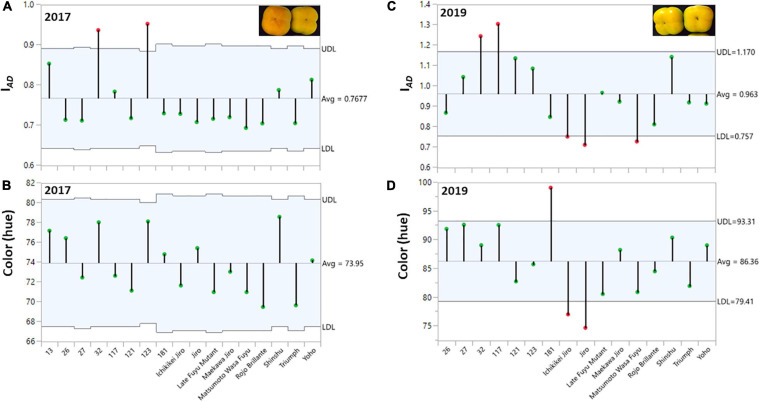
Distribution of chlorophyll (I*_*AD*_*), and color (hue) among the fruits of the persimmon cultivars in two harvests. The ANOM analysis was performed on harvested fruit. **(A,B)** Hue and I*_*AD*_* for harvest 2017; **(C,D)** Hue and I*_*AD*_* for harvest 2019. The green dots represent cultivars within the range and red dots represent cultivars with a deviation from the range at the statistical significance of *p* ≤ 0.001 for harvest 2017 and 2019.

In the persimmon collections of ARO, Israel, there are 7 non-astringent of Japanese origin and 10 astringent cultivars of Chinese origin (Supplementary Table 1, Table 1 and Figure 1), and all of them are seedless. The harvest time of all the cultivars was recorded for five consecutive seasons from 2015 to 2019 (Supplementary Table 1). The cultivars in the collection cover early, mid, and late-season, and their harvest last from August to December. Fruit quality parameters were determined for the years 2017 and 2019. Except for cultivar “13,” the fruits of all of the cultivars in 2019 were harvested earlier than those in 2017. The cultivars “117,” “121,” “181,” “Triumph,” “123,” “Ichikikei Jiro,” and “Jiro” were harvested 8–14 days earlier, while the Cvs. “32” and “Late Fuyu Mutant,” “Shinshu,” “Yoho,” and “Maekawa Jiro” were harvested 17–22 earlier and the cultivars “26,” “Rojo Brillante,” “27,” and “Matsumoto WasaFuyu,” were harvested 25–41 days earlier. Hence, the average hue of all cultivars in 2017 was 73.95 ± 0.43 (less green) and that of 2019 was 85.01 ± 0.74 (with Cv. “13,” and was 86.36 ± 0.65 without Cv. “13”) (more green) (Figure 2).

**TABLE 1:** Size, astringency, TSS, and TA of persimmon cultivars fruit at harvest.

Cultivars type	Cultivars	Diameter (cm)	Height (cm)	Weight (gr)	Astringency index (0–5)	TSS (Brix°)	TA (Malic Acid eq%)
**Astringent**	32	5.80 ± 0.11	4.86 ± 0.12	113.6 ± 8.8	4.8 ± 0.20	23.6 ± 1.1	0.45 ± 0.03
	117	6.12 ± 0.10	5.30 ± 0.20	150.6 ± 6.4	3.6 ± 0.24	25.3 ± 0.5	0.22 ± 0.01
	121	5.40 ± 0.12	4.26 ± 0.08	95.0 ± 6.7	4.2 ± 0.20	29.5 ± 1.0	0.01 ± 0.00
	181	5.36 ± 0.11	4.10 ± 0.14	94.5 ± 5.7	3.6 ± 0.24	21.5 ± 0.8	0.01 ± 0.00
	Triumph	6.38 ± 0.17	4.54 ± 0.14	151.8 ± 14.6	4.8 ± 0.20	28.6 ± 1.1	0.02 ± 0.00
	26	5.32 ± 0.17	3.68 ± 0.05	70.2 ± 2.9	4.6 ± 0.24	18.9 ± 2.5	0.16 ± 0.02
	Rojo Brillante	6.82 ± 0.28	6.96 ± 0.13	216.0 ± 18.5	4.8 ± 0.20	18.3 ± 2.2	0.01 ± 0.00
	27	5.78 ± 0.19	5.22 ± 0.14	137.8 ± 11.9	4.4 ± 0.24	24.5 ± 0.1	0.16 ± 0.01
	13	5.12 ± 0.21	4.02 ± 0.22	77.5 ± 8.5	5.0 ± 0.00	27.2 ± 2.1	0.13 ± 0.00
	123	5.70 ± 0.39	4.48 ± 0.06	134.6 ± 5.1	4.6 ± 0.24	22.7 ± 0.5	0.26 ± 0.01
**Non-astringent**	Matsumoto WasaFuyu	5.02 ± 0.14	3.32 ± 0.13	61.4 ± 6.7	0.4 ± 0.24	23.1 ± 2.1	0.01 ± 0.00
	Late Fuyu Mutant	5.82 ± 0.02	4.16 ± 0.18	103.0 ± 6.0	0.4 ± 0.24	24.6 ± 0.2	0.01 ± 0.00
	Shinshu	6.34 ± 0.15	5.28 ± 0.08	147.7 ± 9.6	0.6 ± 0.24	20.9 ± 1.2	0.07 ± 0.00
	Ichikikei Jiro	6.14 ± 0.14	4.00 ± 0.11	116.4 ± 9.9	0.2 ± 0.20	18.8 ± 0.1	0.06 ± 0.01
	Yoho	5.34 ± 0.13	3.36 ± 0.10	75.0 ± 6.4	0.2 ± 0.20	21.0 ± 1.8	0.09 ± 0.01
	Maekawa Jiro	6.22 ± 0.09	4.02 ± 0.10	124.4 ± 5.8	0.2 ± 0.20	22.4 ± 1.4	0.08 ± 0.01
	Jiro	5.58 ± 0.12	3.88 ± 0.17	97.6 ± 4.5	0.0 ± 0.00	22.1 ± 0.4	0.01 ± 0.00

*The results show the average of 5 representing fruits of each cultivar at harvest; ±SE. The data are of fruit from 2017.*

The fruit shapes of all persimmon cultivars vary from heart-shaped fruits (Cv. “32”) to peanut-shaped fruit (Cv. “Jiro”) (Figure 1). The spherical variation in persimmons is between oblate (Cv. “Yoho”) and prolate fruits (Cv. “Rojo Brillante”) (Figure 1). The weight of the cultivars was between 61 gr (Cv. “Matsumoto WasaFuyu”) to 216 gr (Cv. “Rojo Brillante”). All the persimmon cultivars except Cvs. “26,” “Rojo Brillante,” and “Ichikikei Jiro” had TSS above 20 Brix° and there was no significant difference between the cultivars. The TSS in astringent cultivars was 24.01 ± 1.20 Brix° and for the non-astringent cultivars 21.84 ± 0.69 Brix° (Supplementary Table 2). Based on the titratable acidity (TA) the cultivars can be divided into those which had TA below 0.01 (Cvs. “121,” “181,” “Rojo Brillante,” “Matsumoto WasaFuyu,” “Late Fuyu mutant,” and “Jiro”) those around 0.1–0.2, and only the cultivar “32” had a TA of 0.45. Nevertheless, the astringent group had higher acidity than non-astringent cultivars but it was not significant (Table 1 and Supplementary Table 2).

**FIGURE 3:**
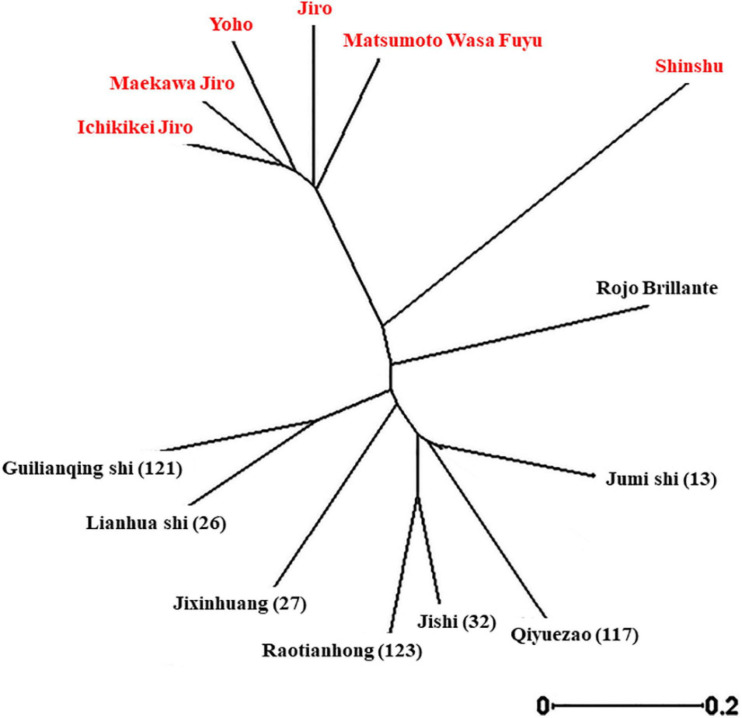
Unrooted neighbor-joining tree of *D. kaki* cultivars using SSR markers.

To investigate the genetic relationships of the different cultivars (Supplementary Table 1), a dendrogram tree was constructed based on UPGMA (Figure 3). This revealed that although Cvs. “Shinshu” and “Rojo Brilliante” are separated from the two main groups, still, all the non-astringent Japanese cultivars examined belong to one group and the other group includes all the astringent Chinese cultivars.

### Comparison of the Cultivar Chlorophyll Content (I*_*AD*_* Index) and Color (Hue) at Harvest

Evaluation of fruit quality parameters from different cultivars requires that the fruit of all cultivars will be at a similar physiological/developmental stage/age. In this study, we determined the chlorophyll (I*_*AD*_*) and color (hue) of the persimmon cultivars for the harvest of two years (2017 and 2019). ANOM analysis was performed to determine the similarity of I*_*AD*_* or hue at harvest among all the cultivars. This analysis is suitable to examine the variability among each cultivar and between the cultivars (Mendeş and Yiğit, 2018). In this test, each vertical line represents the data of all individual fruits for each cultivar (Figure 2), and the average for all the cultivars was marked with a horizontal line. The green dots represent cultivars within the range and red dots represent cultivars with a deviation from the range at the statistical significance of *p* ≤ 0.001 for harvest 2017 and 2019.

**FIGURE 4:**
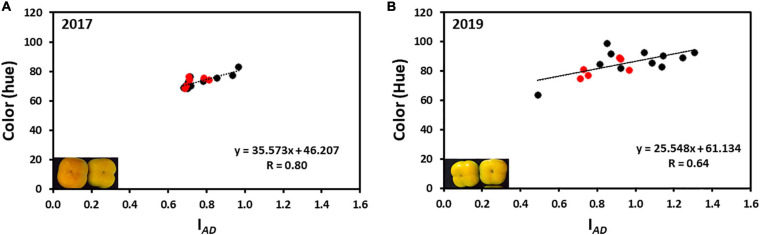
Correlation between chlorophyll (I*_*AD*_*) and color (hue) of persimmon cultivars for harvests of 2017 and 2019. **(A)** Correlation between hue and I*_*AD*_* of harvest 2017; **(B)** Correlation between hue and I*_*AD*_* of harvest 2019. Red and black dots represent the non-astringent and astringent cultivars, respectively.

The average hue value in the year 2017 was 73.95 ± 0.43 and in 2019 was 86.36 ± 0.65 (without Cv. “13,” and was 85.01 ± 0.74 with Cv. “13”) while the I*_*AD*_* was 0.77 ± 0.01 for 2017 and 0.96 ± 0.01 for 2019 (without Cv. “13,” and was 0.93 ± 0.02 with Cv. “13”), respectively. Only two cultivars “Yoho” and “123” were out of the I*_*AD*_* range of average in the year 2017 and when hue was analyzed, all cultivars fall within the average value. However, in the harvest 2019 five cultivars were not included in the average range; Cvs. “32,” and “117” were above the average, while Cvs. “Ichikikei Jiro,” “Jiro,” and “Matsumoto Wase Fuyu” were below the I*_*AD*_* average (Figure 2). However, fewer cultivars; “181,” “Ichikikei Jiro,” and “Jiro,” deviated from the average value of the hue. Interestingly, for harvest 2019 the I*_*AD*_* of Cv. “181” was slightly below the average while the hue was higher than the average and deviated from the collective average. Altogether, the data showed that the parameter of I*_*AD*_* had higher variability than hue. Figure 4 shows the correlation between I*_*AD*_* and hue for the years 2017 and 2019. In the year 2017, the R (0.80) was higher than in 2019 (0.64). No significant differences were observed between the group of astringent and non-astringent cultivars for I*_*AD*_* and hue in both years (Student t-test; *p* ≤ 0.05; Supplementary Table 2).

**FIGURE 5:**
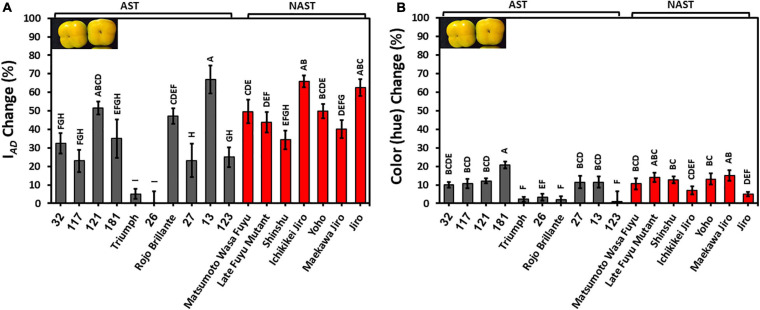
Reduction in chlorophyll (I*_*AD*_*) and color (hue) in persimmon cultivars after 3 months of storage for harvest 2019. **(A)** Change in I*_*AD*_*; **(B)** Change in hue. Bars show the average of 10 fruits in 4 places; ± SE. Different letters indicate a significant difference between treatments according to the Tukey-Kramer HSD test at *p* ≤ 0.05.

The development of color during storage is an important quality trait. I*_*AD*_* for most of the cultivars except Cvs. “Triumph” and “26” were reduced by 20–60%, indicating a reduction in chlorophyll levels. The percent reduction in I*_*AD*_* was significantly lower in astringent cultivars (30.95 ± 5.52) in comparison to the non-astringent (49.46 ± 6.59) (Supplementary Table 2). The hue was also reduced by about 10%, and it was not statistically different between astringent and non-astringent cultivars (Supplementary Table 2). Reduced values of hue and I*_*AD*_* indicate an increase in orange color, for most of the cultivars. Note that the hue of the Cvs. “Triumph,” “26,” and “Rojo Brillante” were hardly modified during storage (Figure 5). The percentage change of I*_*AD*_* was higher than the percentage change of hue, showing major chlorophyll loss during storage.

### Evaluation of Cracks

**FIGURE 6:**
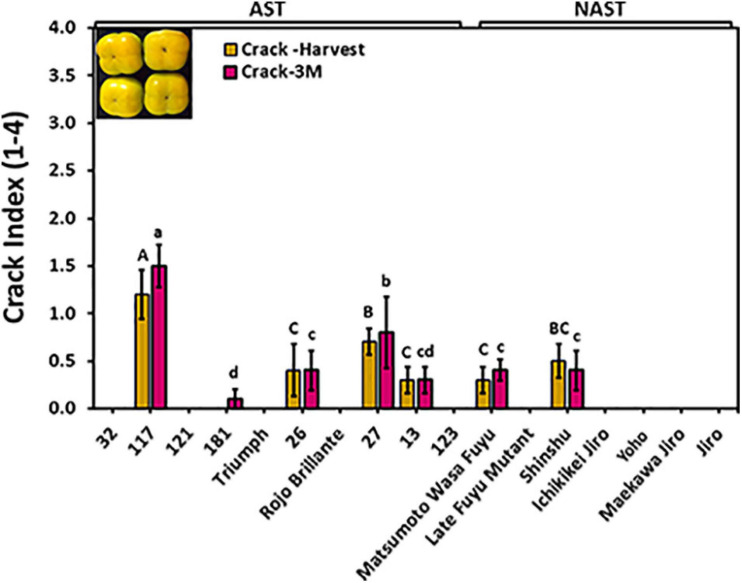
Evaluation of cracks in the different persimmon cultivars. Cracks were evaluated immediately after harvest and following 3 months (3M) of storage. The fruits were dipped in methylene blue and the crack severity was calculated as a crack index. Bars show the average of 5 fruits; ± SE. Different letters indicate a significant difference between treatments according to the Tukey-Kramer HSD test at *p* ≤ 0.05.

Cracks on fruit surfaces reduce their quality, posing a real commercial problem. Cracks were evaluated at harvest time and following 3 months of storage by dipping the fruits of different cultivars for harvest 2019 in methylene blue. Out of 17 cultivars, only 6 cultivars i.e., “117,” “26,” “27,” “13,” “Matsumoto WaseFuyu,” and “Shinshu” had cracks at harvest time, and no significant differences were observed following storage (Figure 6), indicating that most cracks develop in the orchard and not during storage. The cracks index for the majority of the cultivars was below the value of 1 at harvest and after 3 months of storage. However, Cv. “117” developed more severe cracks index that ranged from 1.2 to 1.5. No statistical difference was found for the crack between astringent (0.26 ± 0.13) and non-astringent (0.11 ± 0.08) groups (Supplementary Table 2; Student *t*-test; *p* ≤ 0.05).

### Firmness and Firmness Loss During Storage Is Correlated With Astringency

**FIGURE 7:**
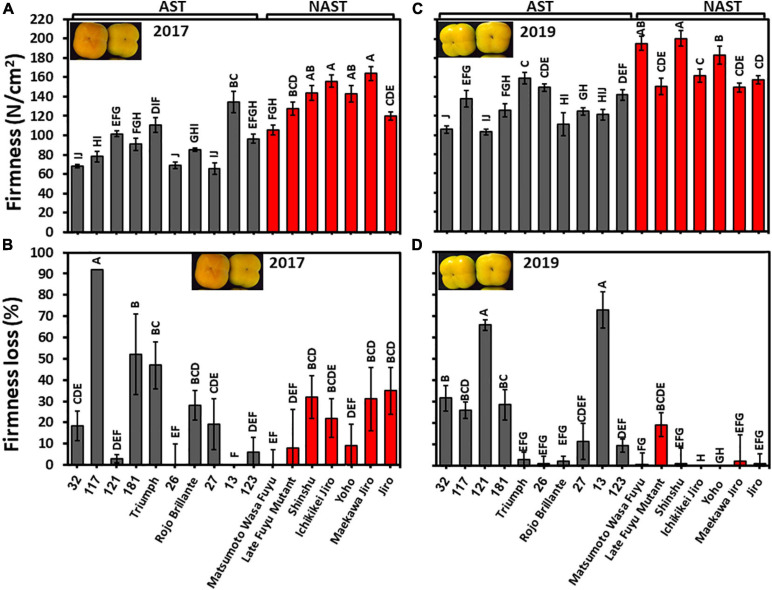
Firmness at harvest and firmness loss following 3 months of storage. **(A)** Firmness of harvest 2017 and **(C)** for harvest 2019. **(B)** Firmness loss of harvest 2017 and **(D)** for harvest 2019. Firmness loss was determined following 3 months of storage at 0°C. Bars show the average of 5 fruits in 2 places; ±SE. Different letters indicate a significant difference between treatments according to the Tukey-Kramer HSD test at *p* ≤ 0.05.

Fruits of all persimmon cultivars of harvests 2017 and 2019 were analyzed for firmness at harvest time and following three months storage. Penetrometer measurements revealed significant differences between the cultivars in both years. Nevertheless, the fruits of harvest 2019 were more firm than the harvest 2017 (Figure 7). The firmness average of astringent/non-astringent, respectively, in the year 2017 was 89.9/137.02 N/cm^2^ and in the year 2019 was 128.94/171.12 N/cm^2^. Hence, the group of non-astringent cultivars was significantly firmer than the group of astringent cultivars for both years (Supplementary Table 2; Student *t*-test; *p* ≤ 0.05).

The cultivars exhibited a significant statistical difference between their firmness losses. Few cultivars of harvest 2017 remained firm (Cvs. “121,” “26,” “13,” “123,” “Matsumoto WasaFuyu,” and “Yoho”) (Figure 7), while more cultivars of harvest 2019 remained firm i.e., “Triumph,” “26,” “Rojo Brillante,” “Matsumoto WasaFuyu,” “Shinshu,” “Ichikikei Jiro,” “Maekawa Jiro,” “Jiro,” and “Yoho” (Figure 7). Firmness loss was significantly higher in astringent cultivars (24.2 ± 8.4%) than in the non-astringent cultivars (14.0 ± 0.8%) of harvest 2019 (Supplementary Table 2; Student *t*-test; *p* ≤ 0.05). Hence, most of the Japanese cultivars remained firmer than the Chinese cultivars if they were harvested at an earlier maturation stage as occurred in 2019. The fruit of the cultivar “121” and “13” lost their firmness in 2019 more than the other cultivars and more than the same cultivars at 2017 because Cv. “13” was more mature at 2019 than in 2017, however, Cv. “121” was slightly less mature in 2019 than in 2017 (Figure 4). The underlying mechanism is still unclear.

### *Alternaria* Susceptibility Is Correlated With Astringency

The common commercially cultivated persimmon “Triumph” and “Rojo Brillante” are susceptible to *Alternaria* (Prusky et al., 1981; Palou et al., 2012), but very little information is available for the other cultivar’s susceptibility, to the best of our knowledge.

**FIGURE 8:**
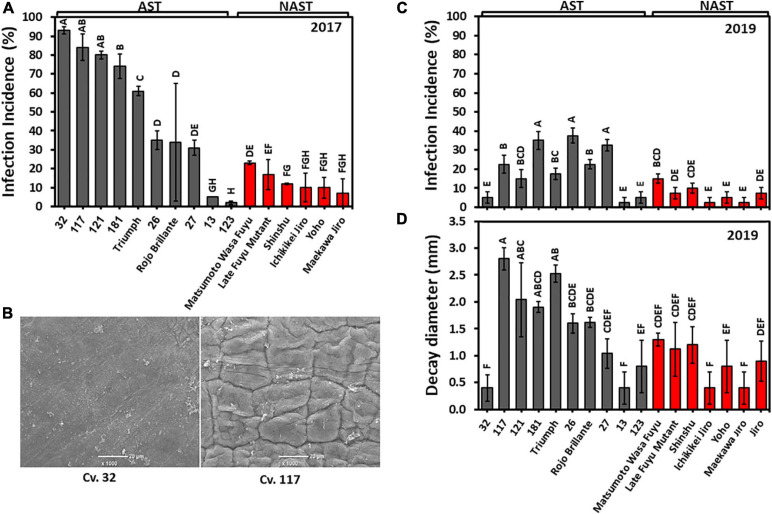
*Alternaria* infection in the different persimmon cultivars in pierced fruit (2017) and non-pierced fruit (2019). **(A)** Infection incidence on pierced fruit for harvest 2017. **(B)** Fruit surface analysis by SEM of Cvs. “32” and “117.” **(C)** Infection incidence on non-pierced fruit for harvest 2019. **(D)** Decay diameter in non-pierced fruit for harvest 2019. Bars show the average of 5 fruits at 8 places; ±SE. Different letters indicate a significant difference between treatments according to the Tukey-Kramer HSD test at *p* ≤ 0.05.

We used two methods to evaluate the sensitivity of the cultivars against *Alternaria*. In the year, 2017 fruits were pierced on the peduncle side, and inoculated with a suspension drop of *Alternaria* spore, while in 2019 the infection was performed on intact skin on the same side. This enabled us to evaluate the role of the skin in infection. The inoculated pierced sites on the fruit of the harvest 2017 developed a higher incidence of *Alternaria* infection than that developed on the non-pierced fruit of 2019 (Figures 8A,C). Nevertheless, in both years the infection patterns in astringent and non-astringent cultivars were very similar and were higher in astringent than in non-astringent cultivars. The most severe infection incidence at 2017 was for pierced Cv. “32” and reached about 90%, while the infection incidence of the same cultivar without piercing was less than 5% (Figures 8A,C). This cultivar is unique among the astringent cultivars in its lower infection rate and in its smaller decay diameter (Figure 8D). SEM analysis of the surface revealed the major differences between Cv. “32” and another Cv. “117,” which is prone to cracks (Figure 8B). The surface of Cv. “32” was smooth, while that of Cv. “117” exhibited many crevices. Cvs. 13 and 123 were unique among the astringent cultivars and they exhibited low infection rate in both years (Figures 8A,C). The average infection incidences in pierced fruits for astringent/non-astringent cultivars was 51.9/13.0% for 2017 and in non-pierced fruits was 21.4/7.1% for 2019, respectively (Supplementary Table 2; Student *t*-test; *p* ≤ 0.05).

The difference between astringent and non-astringent cultivars was obvious also when the decay size of all the astringent/non-astringent cultivars was averaged for each cultivar. The average decay size of astringent cultivars without Cvs. “32” and “13” was 1.79 ± 0.24 mm and that of the non-astringent was 0.87 ± 0.13 mm (Supplementary Table 2; Student *t*-test, *p* ≤ 0.05).

### Correlation and Clustering Analyses Between the Fruit Quality Traits

To further compare the astringent and the non-astringent cultivars, a heat map which include all the traits was created for the years 2017, and 2019 (Supplementary Figure 1). This analysis showed that there were two clades which one included most of the Chinese astringent cultivars and the other most of the Japanese non-astringent ones. This shows that by in large, there are major differences between the phenotypic traits of astringent and non-astringent cultivars in two separate years. The firmness of both years was highly correlated (*R* = 0.52; *p* ≤ 0.05). Comparing parameters of astringent and non-astringent cultivars (Supplementary Table 2) revealed that the parameters including astringency, I*_*AD*_* change, firmness, firmness loss, infection incidence, and decay diameter were statistically different between astringent and non-astringent cultivars in both years for most of the traits.

Table 2 describes the Pearson correlation coefficient between the different parameters of harvest 2017 and 2019. This table shows high negative correlations between astringency and firmness (−0.72) and between firmness and infection incidence (−0.63) for the year 2017. The negative correlation between firmness and infection incidence existed also for the year 2019, but was lower for (−0.22). Nevertheless, a significant correlation coefficient was observed for firmness at 3 months and decay diameter (−0.57). This emphasized that traits of firmness and *Alternaria* resistance were high in non-astringent cultivars in comparison to astringent cultivars in the two years (Supplementary Table 2). Firmness loss was negatively correlated with firmness at harvest (−0.65), and cracks had a low non-significant correlation with infection incidence (0.41) in non-pierced fruit (harvest 2019).

**TABLE 2:** Pearson correlation coefficients (R) matrix for fruit quality traits among the different harvested persimmon cultivars. **(A)** Harvest 2017. **(B)** Harvest 2019.

A											
Harvest 2017	Ast index (0–5)	TSS (Brix°)	TA (MA eq%)	I*_*AD*_*-H	Color (hue)-H	Firmness-H	Inf inc (%)
Ast index (0–5)	1						
TSS (Brix°)	0.33	1					
TA (MA eq%)	0.43	0.02	1				
I*_*AD*_*-H	0.33	0.08	0.80**	1			
Color (hue)-H	0.20	–0.16	0.72**	0.80**	1		
Firmness-H	−0.72**	–0.08	–0.44	–0.11	0.02	1	
Inf inc (%)	0.50	0.37	0.27	–0.02	–0.30	−0.63**	1

**B**

**Harvest 2019**	**I*_*AD*_*-H**	**I*_*AD*_* -3M**	**Color**	**Color**	**Crack-H**	**Crack-3M**	**Firmness-**	**Firmness-**	**Firmness**	**Inf inc**	**Dec dia**
			**(hue)-H**	**(hue)-3M**			**H**	**3M**	**loss (%)**	**(%)-3M**	**(mm)-3M**

I*_*AD*_*-H	1										
I*_*AD*_*-3M	0.82**	1									
Color (hue)-H	0.62**	0.67**	1								
Color (hue)-3M	0.57**	0.77**	0.81**	1							
Crack-H	0.47	0.47	0.27	0.24	1						
Crack-3M	0.48	0.46	0.30	0.24	0.99**	1					
Firmness-H	–0.15	–0.08	–0.01	–0.03	0.08	0.03	1				
Firmness-3M	–0.19	–0.10	0.06	0.16	–0.03	–0.08	0.91**	1			
Firmness loss (%)	0.20	0.06	–0.14	–0.34	0.09	0.12	−0.65**	−0.90**	1		
Inf inc (%)-3M	0.14	0.41	0.59*	0.59*	0.41	0.43	–0.22	–0.18	0.04	1	
Dec dia (mm)-3M	0.35	0.36	0.38	0.25	0.05	0.11	–0.50	−0.57*	0.54*	0.31	1

Ast: Astringency; MA: Malic acid; H: Harvest; Inf: Infection; inc: incidence. H, Harvest; 3M, 3 month; Inf, Infection; inc, incidence; Dec, Decay; dia, diameter. **Correlation is significant at the *p* ≤ 0.01; *Correlation is significant at the *p* ≤ 0.05.

## Discussion

Developing persimmon fruit with high qualities has been a goal in several breeding programs (Yakushiji and Nakatsuka, 2007; Yamada, 2012; Zhang et al., 2016), but postharvest traits were rarely examined. In this study, we evaluated commercially important postharvest traits, in the Israeli persimmon cultivar collection. The genetic analysis of this collection confirmed that the Chinese, astringent cultivars clustered to one group and the Japanese, non-astringent to another group (Figure 3). Cultivar “Triumph” which was not included in this analysis has been demonstrated to belong to a completely different clade, but with some similarity to Rojo Brillante (Yonemori et al., 2008). The traits examined were TSS, TA, firmness and firmness loss during storage, color and color development during storage, cracks development, and susceptibility to a major persimmon pathogen *Alternaria* (Kobiler et al., 2011).

To compare postharvest quality parameters among the different cultivars, with different ripening dates, we have harvested fruits of similar color. The color of persimmon is dependent on the degradation of chlorophyll and the accumulation of carotenoids (Gross et al., 1984), and probably it is indicative of the physiological age. The physiological age was measured by chlorophyll levels (I*_*AD*_*) and hue for all the cultivars (Figure 2). A high correlation was found between I*_*AD*_* and hue (Figure 4 and Table 2). The correlation between color development and I*_*AD*_* index has been demonstrated before in peach (Ziosi et al., 2008), and in two cultivars of persimmon (Kou et al., 2020). Chlorophyll degradation has been shown in melon, to be ethylene sensitive, whereas, carotenoid development is less dependent on ethylene (Pech et al., 2008; Friedman, 2019), suggesting different control pathways. If this is the case for persimmon, it might explain why there was a higher correlation between hue and I*_*AD*_* in the fruits of 2017, which were harvested more mature than for 2019. Different control pathways of chlorophyll degradation and color development may also explain the phenomenon of Cv. “181,” exhibiting a different directional change in chlorophyll levels and hue (Figure 2). Interestingly, although the values of both I*_*AD*_* and hue were higher in the year 2019 than in 2017, three cultivars (“Ichikikei Jiro,” “Jiro,” and “Matsumoto WasaFuyu”) exhibited more advanced phenotype in this year than the rest of the cultivars (Figure 2), suggesting that environment play a role in ripening in these cultivars.

Although both parameters (hue and I*_*AD*_*) are non-destructible and easy to score in large-scale phenotyping, our results suggest that the chlorophyll levels (I*_*AD*_*) can be a better parameter than hue to determine the physiological age of the fruit. This is because higher variability among the fruits of the same cultivar was detected using the I*_*AD*_* than the hue in both years (Figures 2, 4), indicating that chlorophyll degradation is a more rigorous parameter to detect the physiological age. Using chlorophyll levels as a tool for the determination of physiological age has been suggested earlier (Zude, 2003).

Color development during storage is a commercially important trait, especially when fruits are harvested less mature, and it can result from chlorophyll degradation and carotenoid accumulations (Gross et al., 1984). The chlorophyll loss and color development occurred during three months of storage in all the persimmon cultivars (Figure 5). Interestingly, the non-astringent cultivars tended to lose their chlorophyll more than the astringent cultivars and the cause of this is still unclear (Supplementary Table 2). The chlorophyll loss observed here is corroborated by previous transcriptome analysis demonstrating a reduction in the photosynthesis pathway during fruit ripening (Jung et al., 2017).

Since crack development can reduce the fruit quality (Onoue et al., 2016), in this study we found that few of the Chinese cultivars “117,” “26,” “27,” and “13,” while two of the Japanese cultivars “Matsumoto WasaFuyu” and “Shinshu” developed cracks at an early stage of fruit ripening (green-orange stage). In our study, crack development was not correlated to fruit firmness as has been suggested (Onoue et al., 2016), had a low correlation to infection incidence of *A. alternata*, which was determined in non-pierced skin in the year 2019 (Table 2). Hence, it is possible that cracks, which develop in the orchard, can lead to quiescence infection, which develops during storage. Previously, it has been demonstrated that cracks in Cv. “Triumph” increased *Alternaria* infection (Biton et al., 2014). Cracks development at least for Cv. “117” increased with fruit maturity in the orchard, but it did not increase after harvest (Figure 6; Yadav et al., 2021b), although the fruit continues in its ripening program, manifested in color development (Figure 5). The crack-resistance trait exists in the background of the non-astringent cultivars, and following a long-standing effort, a Japanese group has developed a crack-free non-astringent cultivar (Yamada, 2012).

Firmness was lower in the mature fruit (2017) in comparison to the less mature one (2019) and fruit of most cultivars lost their firmness following 3 months of storage at 0°C (Figure 7). Firmness loss has been reported before in persimmon during storage at 0°C (Khademi et al., 2012) and various cell wall degrading enzymes can affect it (Friedman, 2019). The firmness loss can result from differences in any of the genes encoding cell wall degrading enzymes. In persimmon, firmness loss is accompanied by changes in cell wall-derived components (Grant et al., 1992), and pectin which is a major component of the cell wall undergoes depolymerization during ripening (Cutillas-Iturralde et al., 1993). However, no polygalacturonase (PG) activity, which depolymerizes pectin, has been identified in persimmon (Cutillas-Iturralde et al., 1993) and in another study, no change during maturation has been detected in “Rojo Brillante” (Salvador et al., 2007). In another cultivar “Fuping Jianshi” the PG, pectin lyase (PL), pectinesterase (PE), and endo β-gluconase (EGase) increased during ripening (He et al., 2018). In contrast, a novel xyloglucan endotransglucosylase hydrolase (*DkXTH8*) which can be involved in cell wall degradation has been also discovered for involvement in postharvest softening of persimmon (Han et al., 2016).

This study showed that firmness is higher in non-astringent cultivars than in astringent ones in two fruit developmental stages (2017 and 2019), and also firmness loss is higher in astringent cultivars, in comparison to non-astringent cultivars especially in less mature fruit (Supplementary Table 2 and Figure 7). In a different study, the astringent cultivar, “Mopan” was also found to be less firm than the non-astringent cultivar “Yoho,” although “Yoho” was harvested more mature (Kou et al., 2020). In that, study PG and pectin methylesterase (PME) gene expression were lower in “Yoho” than in “Mopan.” As of now, it is not clear why astringent cultivars have lower firmness and they tend to lose their firmness more than non-astringent ones, and the role of cell wall degrading enzymes in these differences has not been elucidated.

In this study, we determined that the genetic modification causing non-astringency also enhanced firmness, *Alternaria* resistance, and reduced-firmness loss during storage (Figures 6, 8 and Supplementary Table 2). As of now, it is not clear if all these traits (astringency, high firmness, and low susceptibility to *Alternaria*) are related to each other and resulted from a pleiotropic effect, caused by a parallel changes in the pathways/compounds in the non-astringent cultivars leading to non-astringency. In our previous study, we suggested that phenolic compounds in non-astringent cultivars contribute to *A. alternata* resistance (Yadav et al., 2021a), but it is not clear if these compounds are also affecting the other traits. Alternatively, the mutation causing non-astringency can cause a defect in an upstream component, which might affect many non-related pathways. Interestingly, a mutation in pectate lyase in tomato caused both higher firmness and higher resistance to the pathogen but the mechanism is still unclear (Yang et al., 2017).

In this study, we demonstrated that the peel might play a major role in *Alternaria* infection. This is supported by the unique results of Cv. “32.” This cultivar was extremely sensitive to *Alternaria* when the infection was done on pierced fruit but was resistant when conidia were placed on the fruit surface (Figure 8). This cultivar is also unique in its higher acidity and higher I*_*AD*_* levels in both years in comparison to the rest of the cultivars although, the hue had an average value (Figure 2 and Table 1).

## Conclusion

Chlorophyll (I*_*AD*_*) can be used to determine the developmental age of persimmon fruit from different cultivars. The use of I*_*AD*_* enabled the comparison of postharvest-related traits of all cultivars at the same developmental age. Astringent cultivars were more sensitive to *Alternaria* infection and less firm than non-astringent cultivars. Few of the cultivars develop cracks already at early developmental age.

## Data Availability Statement

The original contributions presented in the study are included in the article/Supplementary Material, further inquiries can be directed to the corresponding author/s.

## Author Contributions

AY, BK, AF, and HF: study design, acquisition of data, analysis, and interpretation of data. DI, AI, and SZ: resources and methodology. HF and AY: manuscript writing and revising. All authors contributed to the article and approved the submitted version.

## Conflict of Interest

The authors declare that the research was conducted in the absence of any commercial or financial relationships that could be construed as a potential conflict of interest.

## Footnotes

1 http://darwin.cirad.fr/

2 https://biit.cs.ut.ee/clustvis/

## Supplementary Material

Supplementary Figure 1 Hierarchical clustering dendrogram and heat-map among the different parameters examined on the persimmon cultivars. **(A)** Harvest 2017. **(B)** Harvest 2019. Heat maps were constructed by including the normalized parameters of each of the years.

Supplementary Table 1 Harvest dates of persimmon cultivars from 2015 to 2019.

Supplementary Table 2 Differences between astringent and non-astringent fruit quality parameter for the years 2017, 2019. Numbers presents the averages of each trait for all cultivars and the significance of the difference was performed by *t*- test at *p* ≤ 0.05 analysis.
