# Supplementary figures and images for: Phenotypic Characterization of Postharvest Fruit Qualities in Astringent and Non-astringent Persimmon (Diospyros kaki) Cultivars
Source: Front Genet. 2021 Jun 7;12:670929. doi: 10.3389/fgene.2021.670929 (PMC8215578; doi:10.3389/fgene.2021.670929)

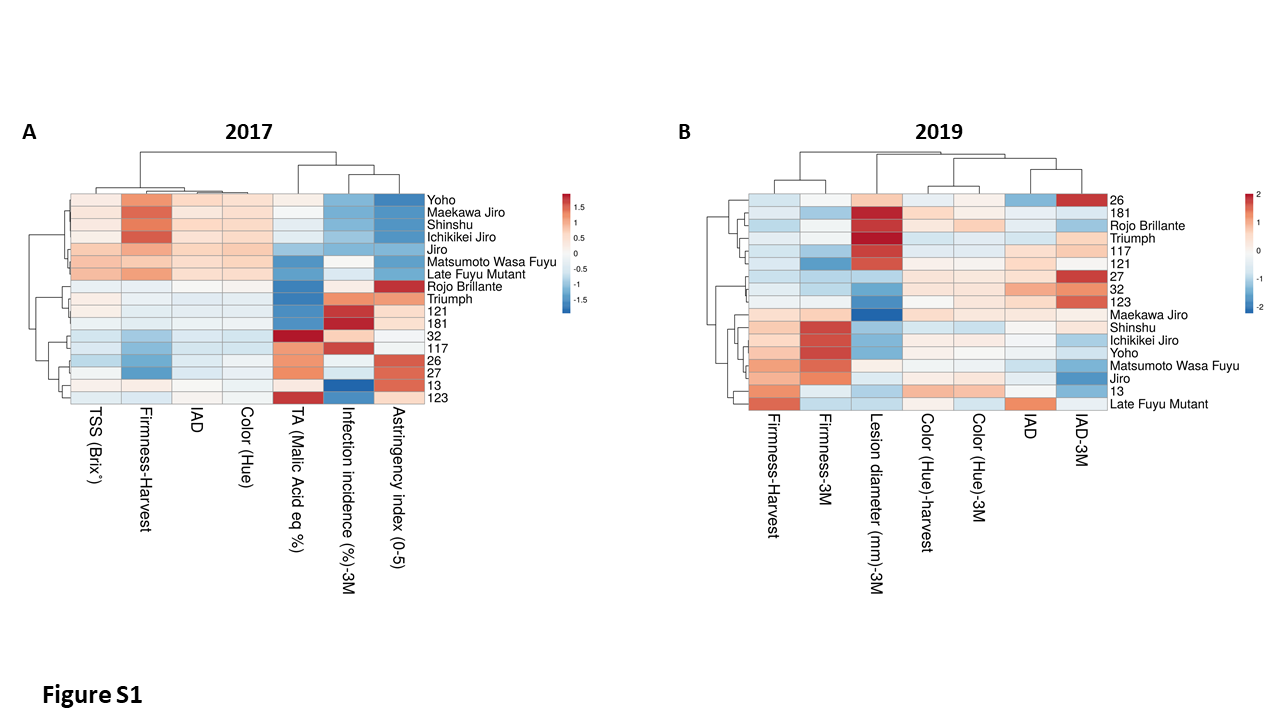

Supplement: Supplementary Figure 1 — Hierarchical clustering dendrogram and heat-map among the different parameters examined on the persimmon cultivars. (A) Harvest 2017. (B) Harvest 2019. Heat maps were constructed by including the normalized parameters of each of the years. [file Image_1.TIF]
