# Supplementary material for: Phenotypic Characterization of Postharvest Fruit Qualities in Astringent and Non-astringent Persimmon (Diospyros kaki) Cultivars
Source: Front Genet. 2021 Jun 7;12:670929. doi: 10.3389/fgene.2021.670929 (PMC8215578; doi:10.3389/fgene.2021.670929)
Supplement: Supplementary Table 1 — Harvest dates of persimmon cultivars from 2015 to 2019. [file Table_1.docx]

**Table S1.**

| **Cultivars type** | **Persimmon cultivars number** | **Persimmon cultivars name** | **Harvest date** | | | | |
| --- | --- | --- | --- | --- | --- | --- | --- |
|  |  |  | **2015** | **2016** | **2017** | **2018** | **2019** |
| **Astringent**  **(AST)** | 32 | Jishi | 01/10 | 20/09 | 02/10 | 17/10 | 15/09 |
|  | 117 | Qiyuezao | 14/08 | 07/08 | 29/08 | 14/08; 20/08 | 21/08 |
|  | 121 | Guilianqing shi | 20/11 | 01/12 | 19/11 | - | 05/11 |
|  | 181 | Yuanxiao shi | 26/11 | 01/12 | 19/11 | - | 05/11 |
|  |  | Triumph | 20/10 | 04/11 | 05/11 | - | 10/10; 28/10 |
|  | 26 | Lianhua shi | 20/10 | 20/09 | 02/10 | - | 05/09 |
|  |  | Rojo Brillante | 02/11 | 01/11 | 05/11 | - | 10/10 |
|  | 27 | Jixinhuang | 01/10 | 20/09 | 16/10 | - | 05/09 |
|  | 13 | Jumi shi | 20/10 | 01/11 | 02/10 | - | 06/10 |
|  | 123 | Raotianhong | 10/08 | 14/08 | 29/08 | 12/08; 4/09 | 19/08 |
| **Non-astringent (NAST)** |  | Matsumoto Wasa Fuyu | 02/11 | 01/11 | 05/11 | - | 06/10 |
|  |  | Late Fuyu Mutant | - | 01/12 | 19/11 | - | 28/10 |
|  |  | Shinshu | 21/09 | 20/09 | 02/10 | - | 09/09; 15/09 |
|  |  | Ichikikei Jiro | - | 20/09 | 16/10 | - | 06/10 |
|  |  | Yoho | - | 20/09 | 16/10 | - | 24/09 |
|  |  | Maekawa Jiro | 26/10 | 01/11 | 16/10 | - | 24/09 |
|  |  | Jiro | - | - | 05/11 | - | 28/10 |
