# Supplementary material for: Phenotypic Characterization of Postharvest Fruit Qualities in Astringent and Non-astringent Persimmon (Diospyros kaki) Cultivars
Source: Front Genet. 2021 Jun 7;12:670929. doi: 10.3389/fgene.2021.670929 (PMC8215578; doi:10.3389/fgene.2021.670929)
Supplement: Supplementary Table 2 — Differences between astringent and non-astringent fruit quality parameter for the years 2017, 2019. Numbers presents the averages of each trait for all cultivars and the significance of the difference was performed by t- test at p ≤ 0.05 analysis. [file Table_2.docx]

**Table S2.**

| **S. No.** | **Characteristics** | **Astringent** | **Non-Astringent** |
| --- | --- | --- | --- |
| 1. | Astringency index (0-5) | 4.44±0.15a | 0.28±0.07b |
| 2. | Weight (g) | 124.16±13.80a | 103.64±11.13a |
| 3. | TA (Malic Acid eq %) | 0.14±0.05a | 0.05±0.01a |
| 4. | TSS (Brix˚) | 24.01±1.20a | 21.84±0.70a |
| 5. | I*_AD_* harvest-2017 | 0.78±0.01a | 0.74±0.01a |
| 6. | Color (hue) harvest-2017 | 74.13±0.55a | 73.67±0.67a |
| 7. | I*_AD_* harvest-2019 | 0.98±0.08a | 0.88 ±0.06a |
| 8. | Color (hue) harvest-2019 | 86.43 ±3.04a | 83.02 ± 2.36a |
| 9. | Crack harvest-2019 | 0.26±0.13a | 0.11±0.08a |
| 10. | I*_AD_* change at 3 month (%)-2019 | 30.95±5.52a | 49.46±6.59b |
| 11. | Color (hue) change at 3 month (%)-2019 | 8.46±1.7a | 11.00±2.0a |
| 12. | Firmness-2017 (N/cm^2^) | 89.90±6.87a | 137.02±7.76b |
| 13. | Firmness-2019 (N/cm2) | 128.02±5.92a | 171.12±8.11b |
| 14. | Infection incidence-2017 (%) | 49.90±10.42a | 13.16±2.21b |
| 15. | Infection incidence-2019 (%) | 19.50±4.06a | 7.14±1.68b |
| 16. | Decay diameter-2019 (mm) without Cv. 13 and 32 | 1.79±0.24a | 0.87±0.14b |
| 17. | Decay diameter-2019 (mm) with Cv. 13 and 32 | 1.59±0.22a | 0.87±0.13a |
| 18. | Firmness loss-2017 (%) | 26.55±9.35a | 19.50±5.26a |
| 19. | Firmness loss-2019 (%) | 24.18±8.39a | 14.00±0.81b |
